# Defining and diagnosing glaucoma: a focus on blindness prevention

**Published:** 2022-01-31

**Authors:** Jibran Mohamed-Noriega, G Chandra Sekhar

**Affiliations:** 1 Associate Professor: Department of Ophthalmology, University Hospital and Faculty of Medicine, Autonomous University of Nuevo Leon, Mexico.; 2 Vice Chair: VST Center for Glaucoma, L V Prasad Eye Institute, Hyderabad, India.

**A diagnostic approach that focuses on patients with definite (or clinical) glaucoma optimises the likelihood of preventing visual disability due to this potentially blinding condition.**

The term glaucoma refers to a group of diseases that affect the optic nerve and could potentially lead to irreversible visual loss. Glaucomatous optic neuropathy is the hallmark of all types of glaucoma. It is characterised by deformation of the optic nerve (see Figure 1, page 4), which manifests as diffuse or focal narrowing of the neuroretinal rim and peripapillary retinal nerve fibre layer loss. The type of glaucoma, the severity of the disease, and the risk of blindness can be assessed by carrying out gonioscopy, slit lamp examination, visual field tests, and intraocular pressure (IOP) measurement.

## Definitions of glaucoma

The definition of glaucoma in adults has changed over the years due to changes in our understanding of how glaucoma affects the eye, the technology available, and the reasons why a particular definition was constructed. A current **clinical definition** of glaucoma is: “A characteristic pattern of glaucomatous optic neuropathy (e.g., narrowing of the neuroretinal rim) with corresponding visual field defects.”

Other changes related to glaucoma might be present in some patients before a clinical diagnosis is made. These may include:

- Thinning of the retinal nerve fibre layer or ganglion cell layer, which can be visualised using optical coherence tomography (OCT)
- Other functional changes such as reduced contrast sensitivity and electrophysiological abnormalities.

However, if clinically significant glaucomatous damage has occurred, then optic disc changes are normally visible, and it is very unusual that glaucoma would be diagnosed from visual field or OCT changes without disc features.

In this article, we want to propose an approach to diagnosing adults with glaucoma that is focused on preventing visual disability. With this approach, we distinguish between two groups of patients:

- Patients with **definite** (or **unequivocal**) glaucoma: those who have definite signs of glaucomatous optic neuropathy. They are at imminent risk of visual loss, usually need treatment, and must be monitored.
- Patients with **suspected** (or **equivocal**) glaucoma: those with possible signs of glaucomatous optic neuropathy. They are not at immediate risk of visual loss, at least in the short term, and usually do not need treatment, but can/must be followed up and monitored (depending on the patient).

We propose that attention is focused on patients with **definite glaucoma** as they are at greater risk of blindness and are likely to require monitoring and treatment by more experienced eye care providers.

## Essential investigations

The following investigations are needed in order to determine whether a patient has definite glaucoma requiring treatment, has suspected glaucoma and also for ongoing monitoring.

**Figure:**
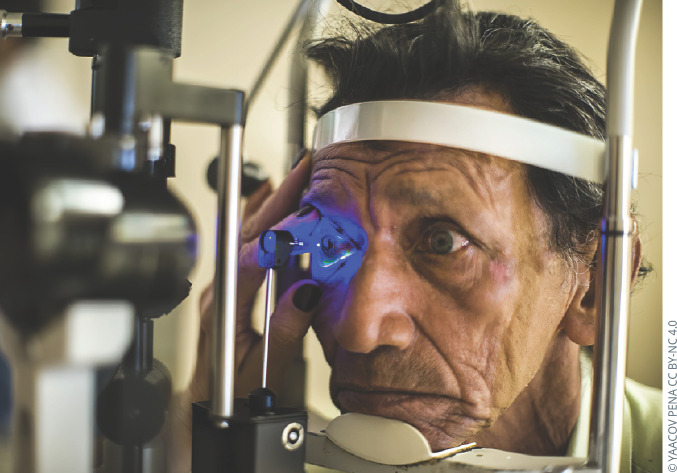
Measuring intraocular pressure using a Goldmann tonometer. **COLOMBIA**

**Visual acuity with best correction.** Although visual acuity only deteriorates at the last stages of glaucoma, measuring it on all visits is important to assess the overall visual function and rule-out other diseases.

**Anterior segment examination using a slit lamp.** This will help in the detection of secondary types of glaucoma such as pseudoexfoliation, uveitic or pigmentary glaucoma.

**Anterior chamber angle assessment** (including gonioscopy). This will determine whether the patient has open-angle or angle-closure glaucoma.

**Examination of the optic nerve head.** This is done using a slit lamp (binocularly) and a 90D or similar lens. Perform a dilated examination to rule out other retinal diseases and to make it easier to examine the optic nerve. Do this on the initial visit and yearly or if any clinical parameter (decreased visual acuity, new onset metamorphopsia etc.) changes significantly.

**Visual field testing.** Carry out static, automated perimetry, commonly performed using a Humphrey visual field machine.

**Tonometry.** Use Goldmann’s applanation tonometer.

## Optional investigations

These additional investigations or tests can be performed if needed and if the equipment is available.

**Measuring central corneal thickness.** This can improve the accuracy of measurements using Goldmann’s applanation tonometer because this method overestimates IOP in patients with thick corneas and underestimates it in those with thin corneas. However, nomograms to ‘correct’ the IOP tend to be inaccurate at an individual level and are not recommended. A thin central cornea might influence the decision if the target IOP is reached in a patient with progressive disease, as the actual IOP is likely to be higher than measured.

**Optical coherence tomography (OCT).** This can help in the examination of the optic nerve and retina. The most frequently used metrics are the average thickness of the circumpapilar retinal nerve fibre and ganglion cell layer. However, each innovation cycle produces a different generation of devices with incompatible measurements, so the results cannot be compared for long-term follow-up assessments.

**Corneal hysteresis.** This non-contact tonometry technique also assesses the corneal biomechanical response and may prove to be helpful for glaucoma assessment.

## Characteristics of definite glaucoma

### After an initial or single examination

The characteristics of a **definite** or unequivocal diagnosis of glaucoma and/or where intervention might be needed, **after an initial or single examination** include:

1. Focal complete loss of the neuroretinal rim. The disc damage likelihood scale (DDLS) is a good way of grading the optic disc.^1^
2. DDLS stage ≥ 6.
3. Cup-to-disc ratio > 0.8.
4. Focal narrowing of the neuroretinal rim, with a corresponding visual field defect. It is important to identify if the sector of the optic nerve that is affected corresponds to the location of the VF defect. Representations of the structure-function relationship, such as the Garway-Heath map, help clinicians identify if the damaged sector of the neuroretinal rim is affecting the corresponding visual field locations (Figure 2).
5. IOP > 35 mm Hg is not diagnostic of glaucoma, but almost all patients with this IOP level need IOP-lowering interventions. A high IOP in the absence of disc damage may be seen in patients with a secondary glaucoma or primary angle closure disease. Over-estimation of the IOP should be considered, e.g., a non-contact tonometer used or a very thick cornea. It is recommended to repeat the measurement before initiating treatment.

**Figure 1:**
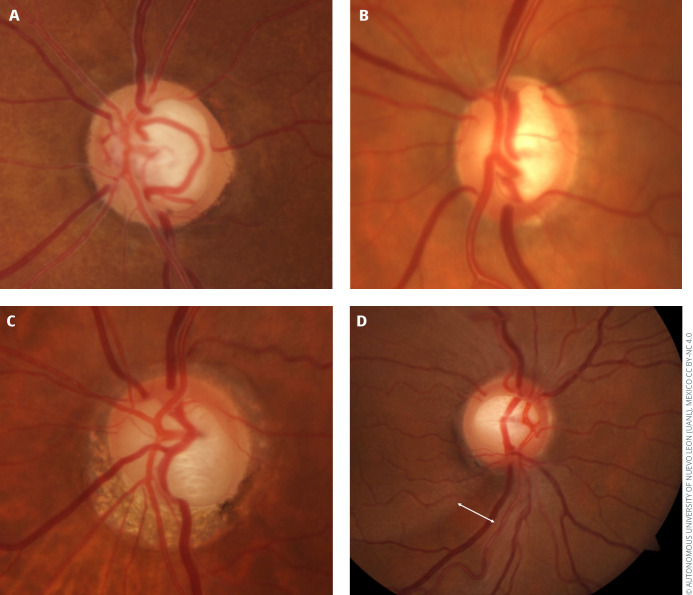
Different forms of glaucomatous optic neuropathy. **A** Enlarged CDR. **B** Focal superior disc rim narrowing. **C** Diffuse loss of the inferior disc rim. **D** Inferotemporal thinning of the disc rim and retinal nerve fibre layer defect.

### After consecutive examinations

On follow-up examination, the following signs of progression would confirm an unequivocal or definite diagnosis of glaucoma:

1. Progression of a **visual field defect** that corresponds to a location of narrow neuroretinal rim or retinal nerve fibre layer loss.
2. Progression of **glaucomatous optic neuropathy:**
  – Enlargement of the vertical cup-disc ratio > 0.2
  – Increase in DDLS > 2 stages
  – Increased narrowing of the neuroretinal rim (change in a sector of the neuroretinal rim (NNR) from narrow to complete loss or from a homogeneous neuroretinal rim to a narrow sector)
  – Significant expansion of a retinal nerve fibre layer defect on OCT
  – Change in the course of vessels due to changes of the optic nerve head
  – The detection of a single new disc haemorrhage usually should not be considered sufficient to diagnose glaucoma or progression, however, they do increase the risk of developing glaucoma and visual field deterioration, particularly if they appear repeatedly.

There are other factors which need to be considered when deciding on and planning the treatment of a person with glaucoma.

**Patients with confirmed glaucoma, i.e., those who are most in need of treatment, can be diagnosed using a slit lamp, tonometer and visual fields alone. Clinicians who do not have access to other instruments, such as OCT, can be reassured that their patients are receiving good care so long as they receive a good clinical examination and visual field analysis.**

## When to suspect glaucoma

The identification of only one of the following characteristics is not enough to confirm the diagnosis of glaucoma, but they do raise the suspicion of possible glaucoma. See Figure 1 for examples.

- Cup-to-disc ratio > 0.7 (this value applies to all disc sizes, but in small discs, a smaller cup-to-disc ratio represents greater damage than in larger discs)
- Diffuse narrowing of the neuroretinal rim
- DDLS stage ≥ 4
- Disc haemorrhages
- Retinal nerve fibre layer defects
- IOP > 24 mm Hg increases the risk for glaucoma, especially in thin corneas
- Abnormal visual field defects (remember that all the diseases that affect the visual pathway from dry eye affecting the cornea, cataracts affecting the lens, retinal changes, to cerebral strokes affecting the posterior visual pathway, can affect the visual field results)
- A reduction in the thickness of OCT parameters.

**It is important to rule out secondary glaucomas which might well carry a high risk of glaucoma blindness.”**

**Figure 2:**
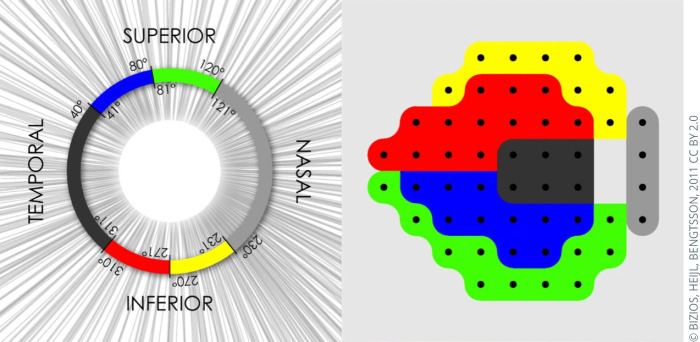
Map representing the relationship between Standard Automated Perimetry visual field sectors and sections of the peripapillary OCT scan circle. This map is based on the work of Garway-Heath et al and shows the correspondence between areas of the visual field and peripapillary retinal nerve fibre layer due to the anatomical configuration of the retinal nerve fibre bundles.^2^

It is important to remember that an OCT scan that flags in red the thickness of the retinal nerve fibre layer, ganglion cell layer, or optic nerve head rim of a patient is not enough to diagnose glaucoma. These parameters are compared to a normative dataset that underrepresent many ethnic and age groups. Similarly, an OCT scan with all parameters in green is not enough to rule out glaucomatous optic neuropathy and should not be considered equivalent to a ‘normal’ optic nerve. OCT changes which, in reality, represent early glaucomatous changes, typically involve only the superior or infero-temporal neuroretinal rim or macular ganglion cell layer. These changes should usually correspond with optic disc changes seen clinically and/or with visual field changes (Figure 2).

It is always important to exclude non-glaucomatous causes of an enlarged cup-to-disc ratio and loss of neuroretinal rim or retinal nerve fibre layers.

## Glaucoma classification

After clinical or definite glaucoma is diagnosed, the two main questions for the clinician are:

### 1. Is this glaucoma primary or secondary?

It is important to rule out secondary glaucomas which might well carry a high risk of glaucoma blindness. These include those which could have causative treatment if identified appropriately (e.g., neovascular glaucoma) and those associated with medical conditions (e.g., uveitic or increased episcleral pressure glaucoma) and which may even potentially be life threatening (e.g., rheumatological diseases and cavernous-carotid fistulas).

### 2. Is the anterior chamber angle open or closed?

Gonioscopy is critical.

The management plan of a patient with glaucoma differs significantly depending on whether the iridotrabecular angle (where aqueous drains out of the eye) is open, narrow or closed and it is vitally important to determine this with gonioscopy.

When a patient is diagnosed with definite glaucoma, it is the responsibility of all eye care providers to advise patients and family about the increased risk of glaucoma in first-degree relatives.
